# Supplementary figures and images for: PTEN knockout using retrogradely transported AAVs restores locomotor abilities in both acute and chronic spinal cord injury
Source: bioRxiv. 2023 Apr 17:2023.04.17.537179. Preprint. [Version 1] doi: 10.1101/2023.04.17.537179 (PMC10153160; doi:10.1101/2023.04.17.537179)

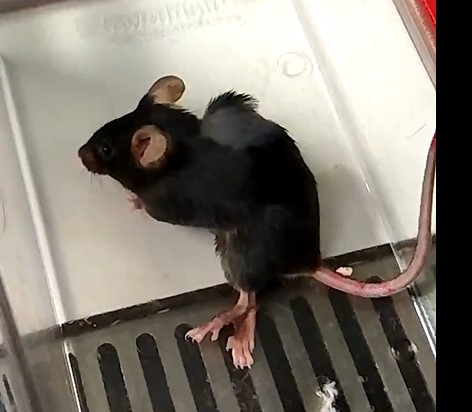

Supplement: Supplement 1 — Supplemental Figure 1. PTEN-KO using AAVrg resulted in sustained contraction of the abdomen. Mice receiving mid-thoracic spinal injections of AAVrg’s to knockout PTEN experienced observable sustained contraction of the abdomen. The extent to which increased abdominal tone and contraction affected weight supporting abilities or trunk stability remains unknown. [file media-1.jpg]
